# miR-30e-3p Promotes Cardiomyocyte Autophagy and Inhibits Apoptosis via Regulating Egr-1 during Ischemia/Hypoxia

**DOI:** 10.1155/2020/7231243

**Published:** 2020-08-17

**Authors:** Bo Su, Xiantao Wang, Yuhan Sun, Manyun Long, Jing Zheng, Wenhao Wu, Lang Li

**Affiliations:** Department of Cardiology, The First Affiliated Hospital of Guangxi Medical University, Nanning, China

## Abstract

**Background:**

Microvascular obstruction (MVO) can result in coronary microcirculation embolism and myocardial microinfarction. Myocardial injury induced by MVO is characterized by continuous ischemia and hypoxia of cardiomyocytes. Autophagy and apoptosis are closely associated with various cardiovascular diseases. Based on our previous study, we observed a decrease in miR-30e-3p expression and an increase in Egr-1 expression in a rat coronary microembolization model. However, the specific function of miR-30e-3p in regulating autophagy and apoptosis in an ischemia/hypoxia (IH) environment remains to be deciphered. We exposed cardiomyocytes to an IH environment and then determined whether miR-30e-3p was involved in promoting cardiomyocyte autophagy and inhibiting apoptosis by regulating Egr-1.

**Methods:**

Cardiomyocytes were isolated from rats for our *in vitro* study. miR-30e-3p was either overexpressed or inhibited by transfection with lentiviral vectors into cardiomyocytes. 3-Methyladenine (3-MA) was used to inhibit autophagy. RT-qPCR and western blotting were used to determine the expression levels of miR-30e-3p, Egr-1, and proteins related to the autophagy and apoptosis process. Autophagic vacuoles and autophagic flux were evaluated using transmission electron microscopy (TEM) and confocal microscopy, respectively. Cardiomyocyte viability was evaluated using the MTS assay. Cell injury was assessed by lactate dehydrogenase (LDH) leakage, and apoptosis was determined by flow cytometry.

**Results:**

Both miR-30e-3p expression and autophagy were significantly inhibited, and apoptosis was increased in cardiomyocytes after 9 hours of IH exposure. Overexpression of miR-30e-3p increased autophagy and inhibited apoptosis, as well as suppressed Egr-1 expression and decreased cell injury. In addition, inhibition of miR-30e-3p reduced autophagy and increased apoptosis and cell injury.

**Conclusions:**

miR-30e-3p may be involved in promoting cardiomyocyte autophagy and inhibiting apoptosis by indirectly regulating Egr-1 expression in an IH environment.

## 1. Introduction

Percutaneous coronary intervention (PCI) is considered an effective myocardial reperfusion strategy for ST-segment elevation myocardial infarction (STEMI). Nevertheless, the occurrence of “no-reflow,” which is a complication caused by a microvascular obstruction (MVO) during PCI, can seriously affect therapeutic efficacy [1–3]. Studies have demonstrated that myocardial injury caused by MVO is characterized by persistent ischemia and hypoxia (IH) of cardiomyocytes after microembolization [4]. However, the molecular mechanisms underlying the regulation of MVO remain to be deciphered.

MicroRNAs (miRNAs) are small noncoding RNAs that modulate posttranscriptional gene expression, including degradation and translational repression [5]. miRNAs are involved in the development of cardiovascular diseases [6], as well as have an important role in alleviating cardiomyocyte injury induced by ischemia or hypoxia [7, 8]. In our previous study, we demonstrated that miR-30e-3p levels, autophagy, and cardiac function were reduced in a coronary microembolization rat model [9]. However, whether miR-30e-3p has a protective or deleterious effect on cardiomyocytes exposed to an IH environment remains elusive.

Autophagy and apoptosis contribute to maintaining cardiomyocyte homeostasis and play a significant role in cardiac physiology [10, 11]. Autophagy and apoptosis interact with each other and are regulated by miRNAs [12, 13]. Autophagy has been demonstrated to alleviate cardiomyocyte injury induced by myocardial ischemia [12]. However, myocardial ischemia can lead to cardiomyocyte apoptosis and myocardial injury [14]. Hence, autophagy plays an important role in cardiomyocyte survival.

Egr-1 is an immediate-early gene and a zinc finger transcriptional protein that has been associated with several cardiovascular diseases [15, 16]. Studies have demonstrated that Egr-1 is regulated by miRNAs in several cardiovascular diseases [17, 18]. In our previous study, we demonstrated that Egr-1 expression levels were increased and involved in the regulation of autophagy and apoptosis in a rat model for coronary microembolization [19]. However, whether miR-30e-3p regulates autophagy and apoptosis via the modulation of Egr-1 in IH-exposed cardiomyocytes is yet to be deciphered.

In this study, we established an *in vitro* model using Sprague-Dawley (SD) rat cardiomyocytes exposed to an IH environment to mimic MVO-mediated myocardial injury. Using this model, we investigated the role of miR-30e-3p in regulating Egr-1 expression on autophagy and apoptosis in IH-exposed cardiomyocytes.

## 2. Methods

### 2.1. Cell Culture and Transfection

#### 2.1.1. Cell Culture

The newborn SD rats (1 to 2 days) were supplied by the animal experiment center of Guangxi Medical University (Nanning, China). The protocols of the animal experiment were approved by the Institutional Animal Care and Use Committee of Guangxi Medical University (Approval No. 201901022). Primary neonatal cardiomyocytes were harvested from the ventricles of newborn SD rats after birth based on a previously published protocol [20]. Tissues were digested with 0.04% collagenase II and 0.08% trypsin with occasional stirring. The supernatants were then transferred to a new sterile container and centrifuged at 12000 rpm for 3 min. Cardiomyocytes were then cultured at 5% CO_2_ at 37°C for 1.5 hours to remove fibroblasts. The unattached cells were then transferred to a new culture flask and incubated in high-glucose DMEM (Gibco, USA) with 10% fetal bovine serum (FBS, Gibco) and 1% penicillin-streptomycin (Solarbio, China). Culture media were changed after 36 hours. To replicate an ischemic environment, the culture media were replaced with FBS-free low-glucose DMEM media (Gibco, USA) and then incubated in a hypoxia incubator (HERAcell VIOS 160i, Thermo Scientific, Waltham, MA, USA) at 37°C, saturated with 3% oxygen, 5% carbon dioxide, and 92% nitrogen, as previously described [21].

#### 2.1.2. Lentivirus Transfection

Lentiviral vectors (Hanbio Biotechnology, China) were used to overexpress or inhibit the expression of miR-30e-3p, including miR-30e-3p mimic (MIR), miR-30e-3p antagonist (MIR antagonist), and miR-30e-3p negative control (NC). The lentivirus was transfected into cardiomyocytes by adding directly to the complete medium at 50 multiplicity of infection (MOI). 24 hours after transfection, culture media were replaced with low-glucose DMEM without FBS and the cells were incubated in a hypoxia incubator. 3-Methyladenine (3-MA) was then added to the cardiomyocytes to inhibit autophagy. The cells were pretreated with 5 mM of 3-MA (Sigma, USA) for 2 hours prior to lentivirus transfection, as described previously [22]. Cardiomyocytes from ten newborn SD rats were used in each group.

### 2.2. Cell Viability

Cell viability was determined by the Cell Titer 96® AQueous One Solution Cell Proliferation Assay (Promega, USA) containing 3-(4,5-dimethylthiazol-2-yl)-5-(3-carboxymethoxyphenyl)-2-(4-sulfophenyl)-2H-tetrazolium (MTS) and the electron coupling reagent phenazine methosulfate. Cells were cultured in 96-well plates and incubated for different durations, followed by the addition of a 20 *μ*l detection reagent to each well. After 2 hours, the optical density for each treatment group was measured at 490 nm [23]. Cardiomyocyte viability was determined as a percentage of the optical density of each group compared to the control group.

### 2.3. Cytotoxicity Assay

The cardiomyocytes cultured in 6-well plates were exposed to IH in each time point and, respectively, transfected with lentivirus as previously described after 24 hours of plating. The lactate dehydrogenase (LDH) leakage assay was implemented using the cytotoxicity detection kit (Jiancheng Bioengineering Institute, China) to measure cell injury following the manufacturer's instruction. The absorbance was measured at 450 nm.

### 2.4. Western Blot Analysis

RIPA buffer (Solarbio, China) was used to extract proteins from the cardiomyocytes. The amount of total protein was measured using the bicinchoninic acid (BCA; Beyotime, China) method. Equal amounts of proteins were loaded and run onto a 10% or 12% SDS-PAGE. The electrophoresed proteins (20 *μ*g) were then transferred to a PVDF membrane (Merck Millipore, USA). The membranes were blocked for an hour in 5% fat-free milk with TBS-T at room temperature. Blots were then incubated with primary antibodies for LC3B, p62, Egr-1, cleaved caspase 3, and GAPDH (Abcam, USA) overnight at 4°C. After three washes with TBS-T, the membranes were incubated with a secondary antibody (Abcam, USA) at room temperature for an hour. The intensity of the protein bands was measured using an imaging system (FLUORCHEMFC3, ProteinSimple, USA) with an enhanced chemiluminescence kit (Thermo Scientific, USA). ImageJ software (National Institutes of Health, USA) was used for densitometric analysis. Specific protein levels were normalized to GAPDH levels.

### 2.5. RNA Extraction and RT-qPCR

The TRIzol reagent (TaKaRa, Japan) was used to extract total RNA following the manufacturer's protocol. RNA concentration was determined using the NanoDrop system (Thermo Scientific, USA). The TaqMan Reverse Transcription Kit (TaKaRa, Japan) was used to synthesize specific cDNA for miR-30e-3p and Egr-1. RT-qPCR was performed using the SYBR Green I PCR kit (TaKaRa, Japan) and the ABI PRISM 7500 system (Applied Biosystems, USA). Primer sequences were designed and synthesized by TaKaRa Biotechnology (Dalian, China) (sequences are listed in Table 1). The 2^-*ΔΔ*Ct^ method was used to calculate the relative expression levels of miR-30e-3p and Egr-1 mRNA normalized to U6 and GAPDH levels, respectively.

### 2.6. Transmission Electron Microscopy (TEM)

Cardiomyocytes were incubated in 6-well plates. After two times of elution with PBS, 0.1 M sodium cacodylate buffer with 2.5% glutaraldehyde was promptly used to fix the cells, after which the cells were transferred to 1% osmium tetroxide and let stand at room temperature for one hour. Ethanol at ascending concentrations (50–100%) was used to dehydrate the specimens. Then, the specimens were embedded in Spurr's epoxy resin and cut apart with an ultramicrotome, after which the thin sections (60–80 nm) were mounted on copper mesh grids. After staining with 1% uranyl acetate and lead citrate, the sections were observed using TEM (H-7650, Hitachi, Tokyo, Japan).

### 2.7. mRFP-GFP-LC3 Adenovirus Transfection

Cardiomyocytes were incubated in confocal dishes with the culture medium containing mRFP-GFP-LC3 adenoviruses (Hanbio, China) at 50 MOI for 2 hours. 24 hours later, the transfection medium was replaced. The cells were then cultivated in FBS-free low-glucose DMEM with hypoxia for subsequent experiments. The dots representing autophagy were measured using a confocal microscope (NIKON, Tokyo, Japan). Quantifying RFP, GFP, and merged points (dots/cell) were used to evaluate autophagic flux.

### 2.8. Flow Cytometry

The Annexin V-FITC/PI double staining kit (BD Biosciences, USA) was used to detect cardiomyocyte apoptosis by flow cytometry. Briefly, cardiomyocytes were collected after IH exposure, washed with ice-cold PBS, and resuspended in 500 *μ*l binding buffer. And then, the cells were incubated with Annexin V-FITC and PI (5 *μ*l each) for 15 min at room temperature while avoiding light. Data were collected with a flow cytometer (BD Biosciences, USA) within one hour and analyzed with the FlowJo software (BD Biosciences, USA).

### 2.9. Statistical Methods

All the continuous data were presented with the measurement of the mean ± standard deviation (SD). The variance was analyzed with one-way ANOVA. All the analysis was conducted with SPSS 22.0 (SPSS Inc., USA). The significance criteria were *P* < 0.05.

## 3. Results

### 3.1. Expression of miR-30e-3p, Egr-1, and Proteins Associated with Autophagy in IH-Exposed Cardiomyocytes

**Figure 1:**
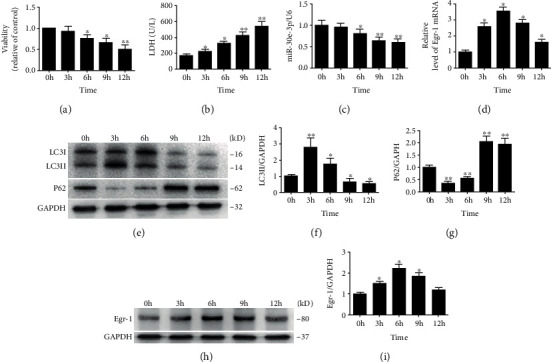
Expression of miR-30e-3p, Egr-1, and key protein involved in autophagy in IH-exposed cardiomyocytes. (a) Viability of IH-exposed cardiomyocytes reduced gradually in a time-dependent manner as determined using MTS assays. (b) Quantitation of LDH secretion levels in IH-exposed cardiomyocytes. (c, d) Expression levels of miR-30e-3p and Egr-1 mRNA measured using RT-qPCR. (e) LC3II and p62 expression determined by western blot analysis. (f, g) Quantification of LC3II and p62 protein levels. (h) Egr-1 protein expression determined by western blot analysis. (i) Quantification of Egr-1 protein levels (*n* ≥ 3; ^∗^*P* < 0.05 and ^∗∗^*P* < 0.01, compared to the 0 h group). h: hour; LDH: lactate dehydrogenase.

Cardiomyocyte activity gradually declined in a time-dependent manner determined using an MTS assay after IH exposure (Figure 1(a)). Cellular viability decreased to 50% in the 12-hour group, suggesting that cardiomyocyte viability reduced in an IH environment. In addition, we found that IH exposure increased LDH levels in cardiomyocytes in a time-dependent manner (Figure 1(b)). This indicated that IH exposure induced cardiomyocyte injury. RT-qPCR was then used to determine the expression of miR-30e-3p and Egr-1 mRNA. miR-30e-3p expression was significantly reduced at 6, 9, and 12 hours of culture (Figure 1(c)). Egr-1 mRNA expression was significantly increased at 3, 6, and 9 hours in the different groups (Figure 1(d)). LC3 and P62 expression, two key proteins involved in autophagy, was measured using western blot analysis (Figure 1(e)). LC3II protein expression was increased significantly in the 3-hour group and then subsequently decreased drastically at 9 and 12 hours (Figure 1(f)). p62 protein levels were reduced markedly at 3 hours and increased significantly after 6 hours (Figure 1(g)). Egr-1 expression was increased significantly at 3, 6, and 9 hours of culture and then decreased after 9 hours (Figures 1(h) and 1(i)). These results indicated that IH exposure downregulated the expression of miR-30e-3p and upregulated Egr-1 expression. In addition, IH exposure inhibited autophagy and induced cardiomyocyte injury.

### 3.2. Observation of Autophagosomes by TEM and Measurement of Autophagic Flux by Confocal Microscopy

**Figure 2:**
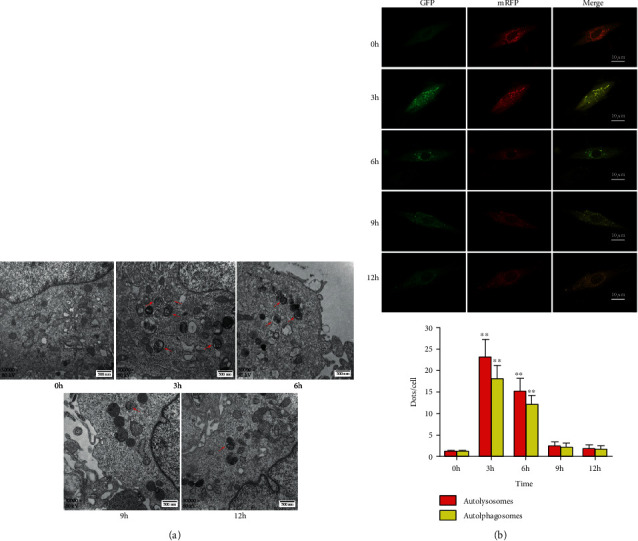
Autophagic vacuoles and autophagic flux determined by TEM and confocal microscopy. (a) Autophagic vacuoles measured using TEM in the five groups. Red arrows represent autophagolysosomes or autophagosomes. Visible intracellular double-membrane autophagic vacuoles and mitochondrial swelling after IH exposure, magnification 30,000x. (b) Autophagic flux determined by confocal microscopy with double fluorescence of mRFP-GFP-LC3. Autophagic flux increased significantly at 3 h post IH exposure and then gradually decreased over time. Magnification 400x (*n* ≥ 3; ^∗^*P* < 0.05 and ^∗∗^*P* < 0.01, compared to the 0 h group). h: hour.

Autophagic vacuole formation is a characteristic indicator of autophagy. During autophagy, intracellular components are encapsulated by double-membrane autophagic vesicles and then fuse with lysosomes to form autolysosomes for subsequent degradation. We observed normal intracellular structures at the baseline; however, after IH exposure, there were visible intracellular double-membrane autophagic vacuoles and mitochondrial swelling. Autophagy was significantly increased at 3-hour IH exposure and then decreased gradually after 6 hours (Figure 2(a)).

Increased levels of autophagosomes or inhibition of autophagosome-lysosome formation leads to LC3 accumulation. Confocal microscopy was used to determine autophagic flux after transfection with mRFP-GFP-LC3 adenovirus. Autolysosomes were represented by the red color and autophagosomes by yellow color (overlay). Autophagic flux demonstrated that autolysosomes (mRFP+dots) and autophagosomes (mRFP+GFP+dots) were increased after 3 hours and reduced afterward in an IH environment (Figure 2(b)).

### 3.3. IH Exposure Induces Apoptosis in Cardiomyocytes

**Figure 3:**
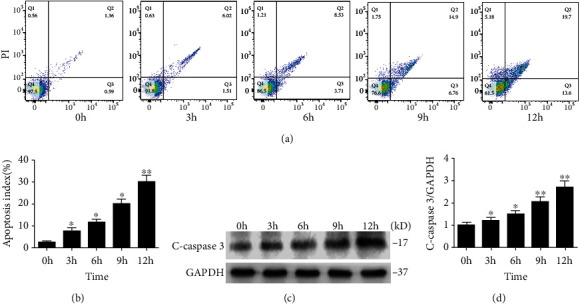
Apoptosis induced by IH exposure in cardiomyocytes. (a) Apoptosis levels of cardiomyocytes measured using flow cytometry. (b) Summarized apoptosis index. (c) Cleaved caspase 3 protein expression levels determined by western blot. (d) Quantification of cleaved caspase 3 protein levels (*n* ≥ 3; ^∗^*P* < 0.05 and ^∗∗^*P* < 0.01, compared to the 0 h group). h: hour; C-caspase 3: cleaved caspase 3.

Apoptosis was determined using flow cytometry and western blot assays. Flow cytometry assays demonstrated that apoptosis in cardiomyocytes increased in a time-dependent manner after IH exposure (Figures 3(a) and 3(b)). IH exposure also increased the levels of cleaved caspase 3, a key apoptosis protein (Figures 3(c) and 3(d)). This indicated that apoptosis increased gradually over time in cardiomyocytes cultured in an IH environment.

### 3.4. Overexpression of miR-30e-3p Promotes Autophagy and Reduces IH-Induced Cardiomyocyte Injury by Regulating Egr-1 Expression

**Figure 4:**
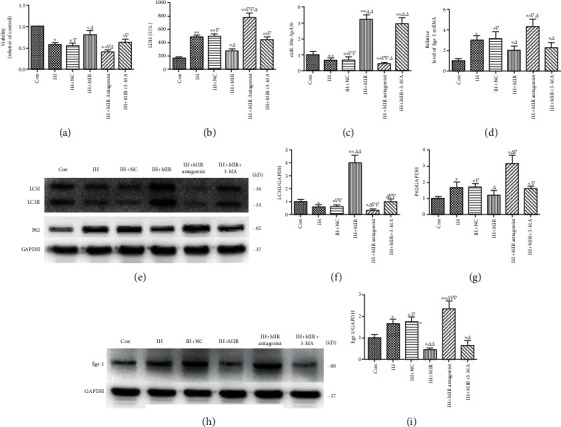
Overexpression of miR-30e-3p promotes autophagy and alleviates cardiomyocyte injury by regulating Egr-1 in IH-exposed cardiomyocytes. (a) Viability of cardiomyocytes determined using MTS assays. (b) Quantitation of LDH secretion levels in IH-exposed cardiomyocytes. The levels of (c) miR-30e-3p and (d) Egr-1 mRNA measured using RT-qPCR. Overexpression of miR-30e-3p reduced Egr-1 mRNA levels, while inhibition of miR-30e-3p increased Egr-1 mRNA expression. (e) Expression of LC3II and p62 determined by western blot. (f, g) Quantification of LC3II and p62 protein levels. (h) Egr-1 expression determined by western blot. (i) Quantification of Egr-1 levels (*n* ≥ 3; ^∗^*P* < 0.05 and ^∗∗^*P* < 0.01, compared to the control group; ^△^*P* < 0.05 and ^△△^*P* < 0.01, compared to the IH group; ^▽^*P* < 0.05 and ^▽▽^*P* < 0.01, compared to the IH+MIR group). Con: control; NC: negative control; IH: ischemia/hypoxia; MIR: miR-30e-3p; 3-MA: 3-methyladenine.

miR-30e-3p expression was increased or reduced using lentivirus transduction of cardiomyocytes. We selected nine hours post IH to evaluate autophagy and cardiomyocyte injury. Cell viability increased significantly, and LDH levels reduced dramatically after overexpression of miR-30e-3p in IH-exposed cardiomyocytes. Conversely, cell viability decreased and LDH levels increased after inhibition of miR-30e-3p (Figures 4(a) and 4(b)). RT-qPCR data demonstrated that miR-30e-3p expression increased and Egr-1 mRNA significantly decreased after overexpression of miR-30e-3p in IH-exposed cardiomyocytes (Figures 4(c) and 4(d)). In addition, LC3II levels increased significantly and p62 and Egr-1 expression was markedly reduced after overexpression of miR-30e-3p. Conversely, after inhibition of miR-30e-3p, Egr-1 and p62 expression increased significantly and LC3II protein was reduced (Figures 4(e) and 4(h)). In addition, LC3II expression was significantly reduced after the addition of 3-MA and miR-30e-3p (Figure 4(e)).

### 3.5. Overexpression of miR-30e-3p Increases Autophagosomes and Autophagic Flux

**Figure 5:**
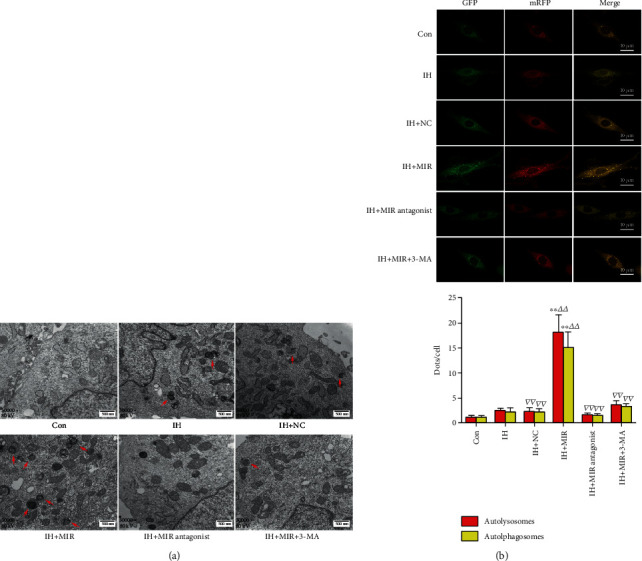
Overexpression of miR-30e-3p augments autophagosomes and autophagic flux. (a) Overexpression of miR-30e-3p promotes autophagy. Autophagic vacuoles measured using TEM; red arrows represent autophagolysosomes or autophagosomes. Increase in the number of autophagic vacuoles after miR-30e-3p overexpression, magnification 30,000x. (b) Autophagic flux determined by confocal microscopy. Overexpression of miR-30e-3p increased autophagy. 3-MA inhibited autophagy in miR-30e-3p overexpressing IH-exposed cardiomyocytes, magnification 400x (*n* ≥ 3; ^∗∗^*P* < 0.01, compared to the control group; ^△△^*P* < 0.01, compared to the IH group; ^▽▽^*P* < 0.01, compared to the IH + MIR group). Con: control; NC: negative control; IH: ischemia/hypoxia; MIR: miR-30e-3p; 3-MA: 3-methyladenine.

Using TEM, the autophagic vacuoles in cardiomyocytes were reduced after exposure to an IH environment. The autophagic vacuoles increased after miR-30e-3p overexpression, while inhibition of miR-30e-3p expression and treatment with 3-MA reduced autophagy (Figure 5(a)). In addition, overexpression of miR-30e-3p significantly increased autophagic flux as determined by confocal microscopy. Conversely, inhibition of miR-30e-3p and treatment with 3-MA reduced autophagic flux (Figure 5(b)).

### 3.6. Overexpression of miR-30e-3p Attenuates Apoptosis in IH-Exposed Cardiomyocytes

**Figure 6:**
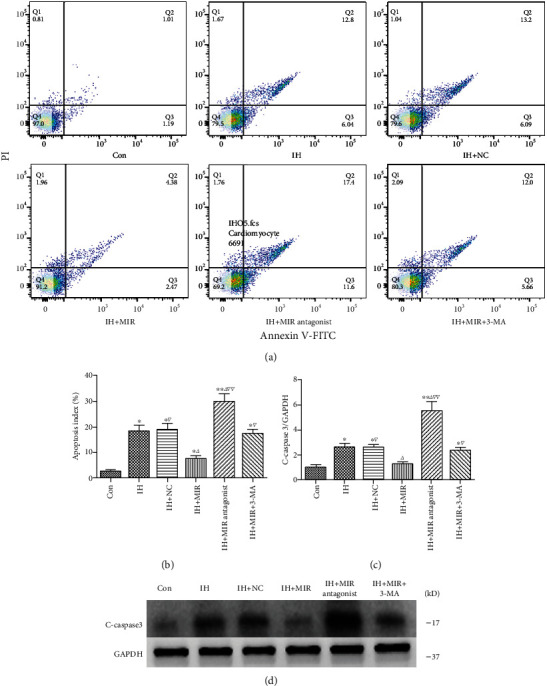
miR-30e-3p overexpression attenuates apoptosis in IH-exposed cardiomyocytes. (a) Apoptosis measured using flow cytometry. (b) Summarized apoptosis indexes. (c) Quantification of cleaved caspase 3 protein levels. (d) Cleaved caspase 3 protein expression levels determined by western blot (*n* ≥ 3; ^∗^*P* < 0.05 and ^∗∗^*P* < 0.01, compared to the control group; ^△^*P* < 0.05 and ^△△^*P* < 0.01, compared to the IH group; ^▽^*P* < 0.05 and ^▽▽^*P* < 0.01, compared to the IH+MIR group). Con: control; NC: negative control; IH: ischemia/hypoxia; MIR: miR-30e-3p; 3-MA: 3-methyladenine; LDH: lactate dehydrogenase; C-caspase 3: cleaved caspase 3.

Flow cytometry assays demonstrated increased apoptosis after cells were treated with a miR-30e-3p antagonist, while miR-30e-3p overexpression significantly reduced apoptosis levels (Figures 6(a) and 6(b)). In addition, cleaved caspase 3 levels were significantly reduced after miR-30e-3p overexpression and increased after miR-30e-3p knockdown (Figures 6(c) and 6(d)). Based on these results, cardiomyocyte apoptosis induced by IH exposure could be inhibited by overexpressing miR-30e-3p.

All these results indicate that miR-30e-3p may be involved in promoting autophagy and inhibiting apoptosis by indirectly regulating Egr-1 expression in IH-exposed cardiomyocytes.

## 4. Discussion

Studies have demonstrated that the characteristic features of myocardial injury induced by MVO are persistent IH of cardiomyocytes after microembolization [4]. In the present study, we cultured rat cardiomyocytes in an IH environment to simulate MVO. We determined that overexpression of miR-30e-3p could promote autophagy and inhibit apoptosis by indirectly regulating Egr-1 expression in IH-exposed cardiomyocytes.

Several studies have demonstrated that cardiomyocyte autophagy is activated during myocardial ischemia [24]. Autophagy provides energy during myocardial ischemia to play a protective role against injury [25]. In addition, inhibition of cardiomyocyte apoptosis improves heart function after ischemia [26]. Our results demonstrated that autophagy in cardiomyocytes increased significantly at 3 hours post IH exposure but decreased gradually soon afterward in a time-dependent manner. This indicated that autophagy increases in response to initial IH but decreases gradually over time with continuous stimulation. Autophagy has been demonstrated to negatively correlate with apoptosis. Reduced autophagy results in an increase in apoptosis and cardiomyocyte cell injury.

miRNAs regulate autophagy and apoptosis by modulating gene expression in cardiomyocytes [27, 28]. miR-30e-3p is a member of the miR-30 family and plays a role in several cardiovascular diseases [29, 30]. Previous studies have demonstrated that members of the miR-30 family play different roles in various environments. miR-30a has been demonstrated to increase autophagy in hypoxia-exposed cardiomyocytes [31], while downregulation of miR-30e could inhibit apoptosis to protect the heart from myocardial ischemia/reperfusion injury [32]. Interestingly, studies have also demonstrated that overexpression of miR-30a inhibits autophagy to alleviate hypoxia/reoxygenation injury in cardiomyocytes [33]. Upregulation of miR-30e-5p has been shown to alleviate hypoxia-induced apoptosis by targeting Bim to protect cardiomyocytes [34]. This suggested that the role of miR-30 family members on cardiomyocyte protection is different under various physiological conditions. In addition, augmentation of autophagy has been shown to protect cardiomyocytes during hypoxia, but excessive activation of autophagy results in cardiomyocyte death during myocardial ischemia/reperfusion injury [35]. Our study demonstrated that IH inhibits miR-30e-3p expression in a time-dependent manner. We observed that autophagy was significantly increased at 3 and 6 hours post IH; however, miR-30e-3p expression levels did not change. This suggests that autophagy was modulated by multiple factors rather than miR-30e-3p alone during the early stages of IH. Upregulation of miR-30e-3p increased autophagy and reduced apoptosis significantly. This was consistent with the increasing trend of autophagic flux and the expression of key autophagic proteins. These results demonstrated that overexpression of miR-30e-3p could promote autophagy and inhibit apoptosis in IH-exposed cardiomyocytes.

In our previous study, we demonstrated that silencing Egr-1 expression could increase autophagy, inhibit apoptosis, and improve cardiac function in a rat model of coronary microembolization [19]. In the present study, we demonstrated that overexpression of miR-30e-3p inhibited Egr-1 expression, as well as augment autophagy, reduced apoptosis, and alleviate cardiomyocyte injury after IH exposure. These were similar to the results of direct inhibition of Egr-1 expression and indicated that Egr-1 expression was negatively correlated with miR-30e-3p expression. This suggested that Egr-1 could be indirectly regulated by miR-30e-3p. Identifying the genes which miR-30e-3p directly regulated involved in the modulation of Egr-1 under IH conditions needs further study.

In this study, we used 3-MA to inhibit autophagy in cardiomyocytes. The results revealed that 3-MA treatment resulted in decreasing autophagy, increasing apoptosis, and aggravating cardiomyocyte injury. It indicated that miR-30e-3p overexpression increasing autophagy can protect cardiomyocytes but not lead to autophagic death and aggravate cardiomyocyte injury. In addition, 3-MA treatment in cardiomyocytes overexpressing miR-30e-3p resulted in increased apoptosis, which supports the antagonistic relationship between autophagy and apoptosis.

## 5. Conclusion

In summary, this study demonstrated that miR-30e-3p serves a significant role in promoting cardiomyocyte autophagy and inhibiting apoptosis induced by IH via indirectly regulating Egr-1 expression. These findings suggest that targeted upregulation of miR-30e-3p expression to increase autophagy during the initial period of IH may alleviate cardiomyocyte injury.

## Figures and Tables

**Table 1:** The sequences of PCR primers used in this study.

Gene	Primer sequences (5′-3′)
miR-30e-3p forward	ACGCTTTCAGTCGGATGTTTACAGC
miR-30e-3p reverse	GTGCGTGTCGTGGAGTCG
Egr-1 forward	GAACAACCCTACGAGCACCTG
Egr-1 reverse	GCCACAAAGTGTTGCCACTG
U6 forward	GGAACGATACAGAGAAGATTAGC
U6 reverse	TGGAACGCTTCACGAATTTGCG
GAPDH forward	GGCACAGTCAAGGCTGAGAATG
GAPDH reverse	ATGGTGGTGAAGACGCCAGTA

## Data Availability

The data used to support the results of this work are available from the corresponding author upon request.
